# Supplementary material for: Genomic transcription factor binding site selection is edited by the chromatin remodeling factor CHD4
Source: Res Sq. 2023 Mar 15:rs.3.rs-2587918. Preprint. [Version 1] doi: 10.21203/rs.3.rs-2587918/v1 (PMC10055546; doi:10.21203/rs.3.rs-2587918/v1)
Supplement: 1 [file NIHPPrs2587918v1-supplement-1.pdf]

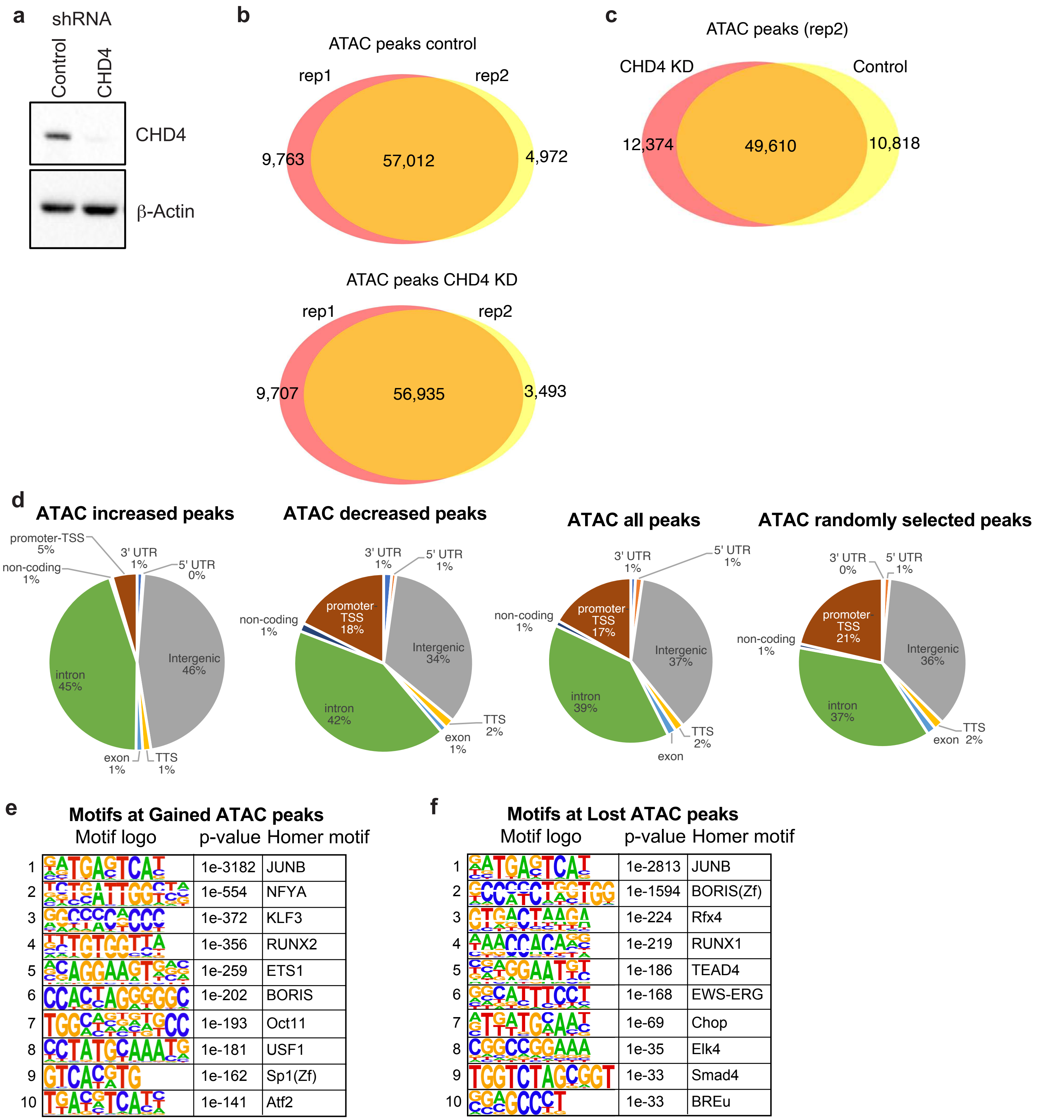

Supplementary Figure 1. ATAC-seq peak overlap in CHD4 knockdown cells

(a) Western blot showing CHD4 knockdown. MDA-MB-231 cells were infected with the lentivirus encoding control shRNA or CHD4 shRNA. β-Actin expression was used as an internal control. **(b)** Venn diagram showing the ATAC-seq peak overlap between biological replicates in control (top) or CHD4 knockdown (KD, bottom) cells. **(c)** Venn diagram showing the ATAC-seq peak overlap between control and CHD4 knockdown MDA-MB-231 cells. **(d)** Pie chart showing peak annotation defined by HOMER. Increased, decreased, all, or randomly selected ATAC-seq peaks are classified into 8 annotation categories. **(e-f)** HOMER de novo motif analysis. ATAC-seq peaks are grouped in Gained (e, uniquely observed in CHD4 knockdown cells) or Lost (f, only observed in the control cells) ATAC-seq peaks (shown in Figure 1b), and are used as input for HOMER motif analysis.

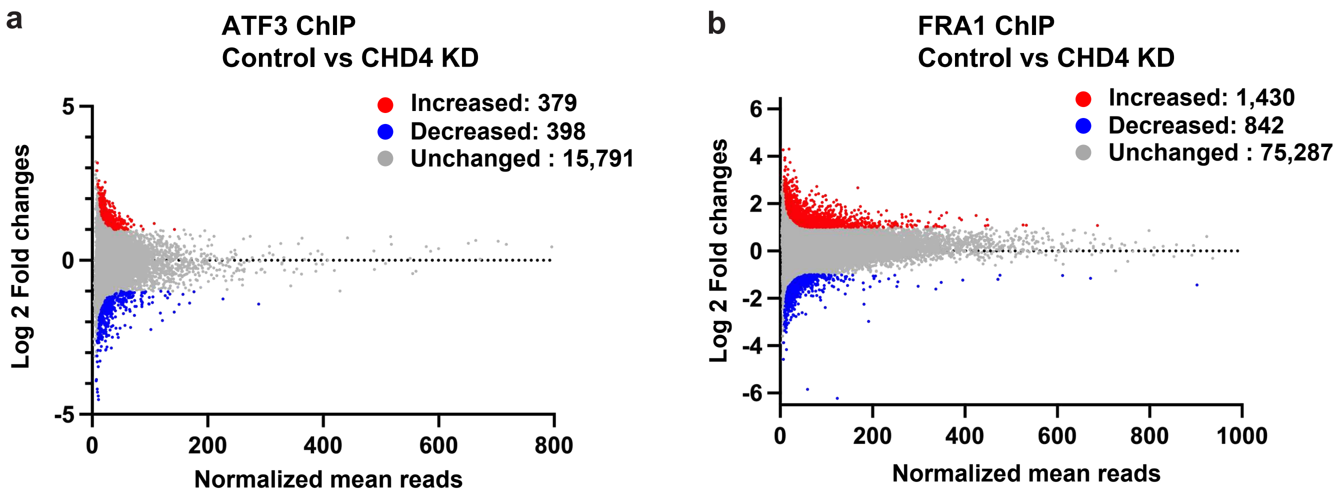

Supplementary Figure 2. Differential peak analyses of ATF3 and FRA1 ChIP-seq data.
(a) Scatter plot shows increased (red), decreased (blue), and unchanged (grey) ATF3 peaks upon CHD4 depletion. **(b)** Scatter plot shows increased (red), decreased (blue), and unchanged (grey) FRA1 peaks upon CHD4 depletion.

Saotome et al. Supplementary Figure 3

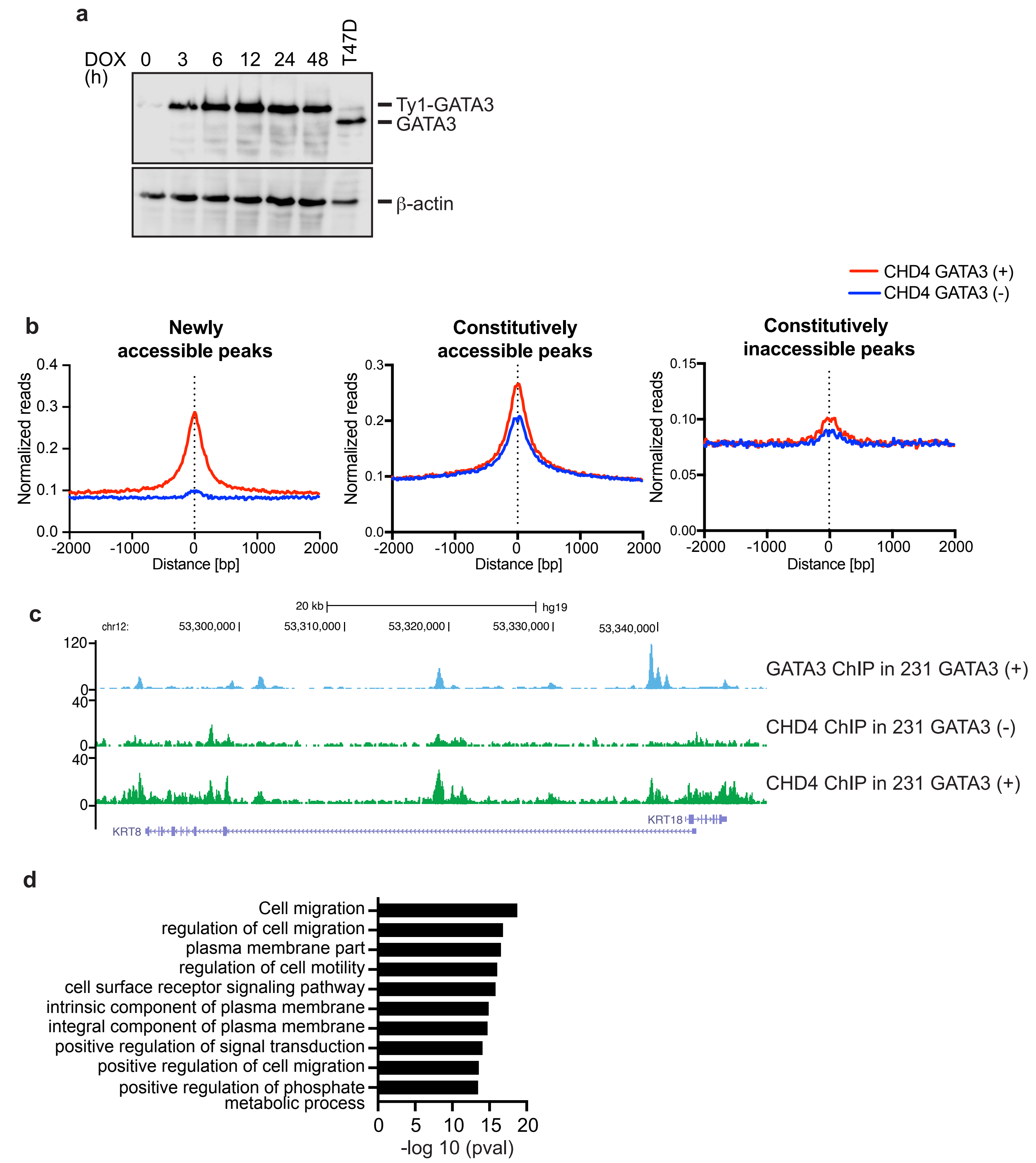

Supplementary Figure 3. De novo motif analysis of GATA3 peaks
(a) Western blot showing GATA3 expression after DOX treatment (1 μg/ml at the final concentration). β-Actin expression was used as an internal control. **(b)** Metaplots showing the CHD4 ChIP-seq signals before and after GATA3 expression. Newly accessible, constitutively accessible. constitutively inaccessible peak groups are previously defined in the GATA3 stably expressed cell system 25. **(c)** Representative genome track of GATA3 and CHD4 ChIP-seq data. GATA3 ChIP-seq was performed in the GATA3-expressed stable cell line. CHD4 ChIP-seq was performed in the GATA3 negative or positive MDA-MB-231 stable cell lines. **(d)** Pathway analysis of the up-regulated genes shown in Figure 4a. Top 10 enriched pathways are shown. expression system.

Saotome et al. Supplementary Figure 4

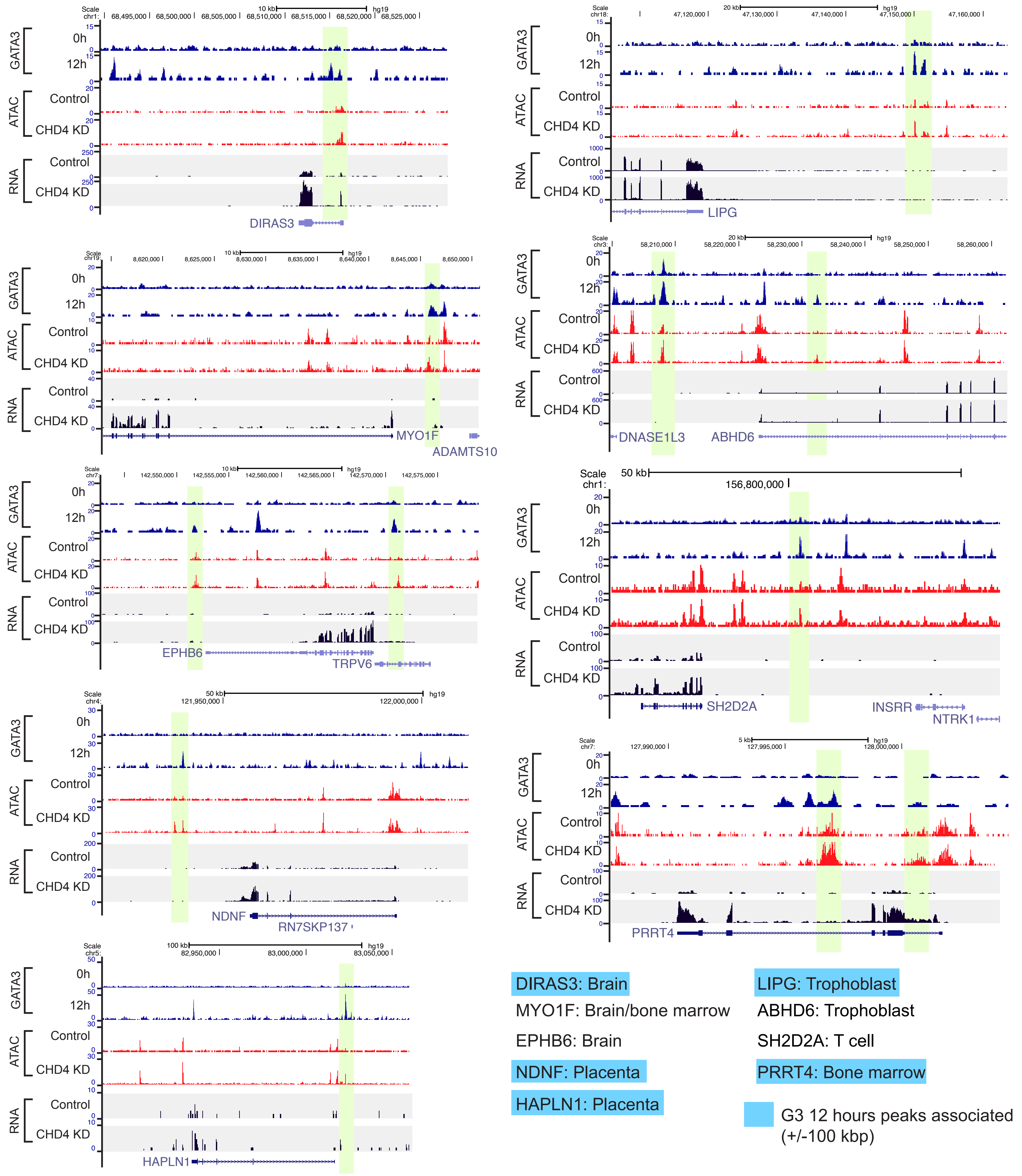

Supplementary Figure 4. CHD4 depletion induces aberrant chromatin opening and gene activation. Genome browser tracks show the examples of aberrant gene expression. Brain, placenta, trophoblast, T cell, and bone marrow related genes are selected. In each figure, GATA3 peaks that have increased ATAC-seq signals in the CHD4 knockdown cells are highlighted. The genes that are associated with the constitutively inaccessible GATA3 peaks (peaks within +/- 100 kbp) were highlighted in light blue.

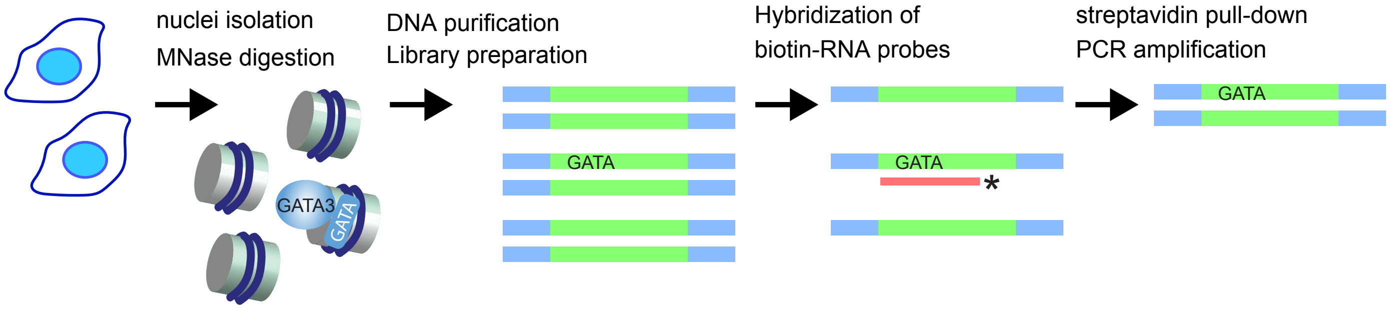

Supplementary Figure 5. Capture MNase-seq
Experimental scheme of capture MNase-seq. Mono-nucleosmal fragments were prepared by MNase digestion. Sequencing libraries were made by NEXTFLEX Rapid DNA-Seq kit (PerkinElmer). Biotinylated RNA probes (Agilent) were used to enrich nucleosome fragments at selected GATA3 peaks.

Supplementary Files

This is a list of supplementary files associated with this preprint. Click to download.

- SupplementaryTable1CaptureMNaseProbes.xlsx
- SupplementaryTable1CaptureMNaseProbes.xlsx
- SupplementaryTable2DEGs.xlsx
- SupplementaryTable2DEGs.xlsx
